# Enhancing adoption of patient safety culture assessments in Brazil: a strategy informed by CFIR and ERIC

**DOI:** 10.1186/s43058-026-00865-7

**Published:** 2026-01-17

**Authors:** Zenewton André da Silva Gama, Magda Machado de Miranda Costa, Heiko Thereza Santana, Natália Gentil Linhares, Evan M. Benjamin, Katherine E. A. Semrau

**Affiliations:** 1 Department of Collective Health, Federal University of Rio Grande do Norte, Natal, RN Brazil; 2 Ariadne Labs, Brigham and Women’s Hospital and Harvard T. H. Chan School of Public Health, Boston, MA USA; 3 Gradate Program in Quality Management in Health Services (QualiSaúde), Federal University of Rio Grande do Norte, Natal, RN, Brazil; 4 Agência Nacional de Vigilância Sanitária, Brasília, DF Brazil; 5 Bachelor of Science in Nursing, Federal University of Rio Grande do Norte, Natal, RN Brazil; 6 Department of Medicine, Harvard Medical School, Boston, MA, USA; 7 Health Policy and Management, Harvard T.H. Chan School of Public Health, Boston, MA, USA

**Keywords:** Patient safety, Organizational culture, Governmental regulation, Hospitals, Implementation science, Brazil

## Abstract

**Background:**

Regular assessments of Patient Safety Culture (PSC) are recommended by the World Health Organization to strengthen healthcare systems. In Brazil, despite national campaigns, hospital adherence to PSC assessments has remained low. This study aimed to design a tailored implementation strategy to improve the uptake of PSC assessments in Brazilian hospitals, addressing the key barriers faced in previous national efforts.

**Methods:**

We conducted a sequential exploratory mixed-methods study in three phases. First, a qualitative survey with 82 patient safety center coordinators identified perceived barriers and facilitators to implementing PSC assessments. Then, a quantitative survey with 297 coordinators prioritized the most relevant barriers. Finally, we used the Consolidated Framework for Implementation Research (CFIR) and the Expert Recommendations for Implementation Change (ERIC) to guide the design of a tailored implementation strategy aligned with the prioritized barriers.

**Results:**

The main barriers included insufficient dissemination of PSC assessments, lack of training for staff, resistance to completing the survey, the excessive length of the questionnaire, and technical limitations of the data collection platform. The co-design implementation strategy includes 16 actions such as improving communication, offering training, adapting the technology platform, and revising roles and responsibilities within hospitals. These actions were aligned with the identified barriers and aim to enhance organizational readiness, reduce complexity, and promote engagement.

**Conclusions:**

Our findings highlight critical factors limiting the adoption of PSC assessments in Brazil and offer a data-driven, context-sensitive implementation strategy to overcome them. These results provide actionable recommendations for policymakers, healthcare managers, and regulators aiming to strengthen patient safety culture in large-scale, resource-constrained health systems.

**Supplementary Information:**

The online version contains supplementary material available at 10.1186/s43058-026-00865-7.

Contributions to the literature

- This study presents a data-driven approach to improve national uptake of patient safety culture assessments in Brazil’s healthcare system.
- It shows how implementation science can support regulatory agencies in redesigning strategies after limited adoption of previous initiatives.
- The findings offer a practical guidance for tailoring large-scale interventions in health systems facing capacity and resource challenges.
- It expands the understanding of how to apply implementation frameworks beyond individual organizations to national public health policies.

## Introduction

Assessing and improving patient safety culture is a global priority to reduce harm in healthcare [1–3]. The World Health Organization (WHO), through the Global Patient Safety Action Plan 2021–2030, recommends that the health systems regularly conduct patient safety culture (PSC) assessments This recommendation is aligned with Strategic Objective 2 of the Action Plan, which aims to build high-reliability health systems and organizations that protect patients from harm on a daily basis. A core indicator of this objective is the number of countries, provinces, or healthcare facilities conducting regular PSC assessments [1].

In Brazil, the Brazilian Health Regulatory Agency (Anvisa) has led efforts to promote PSC assessments at the national level since 2021. The national goal, defined in the Integrated Plan for Patient Safety Management 2021–2025, is to have at least 40% of hospitals with intensive care units (ICUs) conducting PSC assessments by 2025 [4]. Despite two national campaigns (in 2021 and 2023), adherence has remained low, with only about 15% of eligible hospital participating [5, 6]. This persistent gap highlights systemic barriers that limit the adoption of PSC assessments, even when supported by federal initiatives.

Previous attempts to scale up PSC assessments in Brazil were based primarily on dissemination strategies, including webinars, guidance documents, and voluntary calls for participation. However, these efforts lacked structured approaches to identify and address implementation barriers [7]. This reflects a broader challenge faced by national-level interventions in complex health systems: designing strategies that move beyond dissemination to effective, sustained implementation.

Implementation science offers frameworks and tools to guide this process. Among them, the Updated Consolidated Framework for Implementation Research (CFIR) helps identify barriers and facilitators within different domains of the healthcare system [8], while the Expert Recommendations for Implementation Change (ERIC) provides a comprehensive taxonomy of implementation strategies to address those barriers [9].

We selected CFIR and ERIC because they offer a structured approach to identifying contextual barriers and aligning them with targeted implementation strategies. CFIR is a determinant framework designed to capture multilevel factors that influence implementation, while ERIC provides a taxonomy of strategies that can be matched to these barriers [10, 11]. As classified by Nilsen (2015) [12], such frameworks support precision in designing implementation efforts by focusing on determinants rather than outcomes or processes. In contrast, frameworks like RE-AIM or PARIHS emphasize outcome measurement or conceptual relationships, offering less operational guidance for tailoring strategies based on local context.

Evidence from Guatemala, for example, shows how the prospective use of CFIR enabled the identification of barriers and facilitators in a national patient safety program, leading to successful adaptations such as engaging champions and embedding educational activities within error reporting systems [13]. Similarly, in the Veterans Health Administration (VHA), in the United States, the CFIR-ERIC Matching Tool has been used to select strategies based on contextual assessments, with studies showing that strategies aligned with the tool’s recommendations were significantly more effective than those not aligned [14]. In Japan, the retrospective application of CFIR was used to understand and refine the implementation of a patient safety reporting system, highlighting the critical role of matching strategies to contexts to improve results [15].

While these examples demonstrate the value of CFIR and ERIC in both prospective and retrospective applications, their use to design national-level strategies – particularly within middle-income countries and large, resource-constrained health systems like Brazil’s – is still underexplored [16].

This study aims to address this gap by designing a national implementation strategy to improve the uptake of patient safety culture assessments in Brazilian hospitals. Using a sequential mixed-methods design, the study integrates the perspectives of patient safety leaders across the country to identify key barriers and align them with evidence-informed implementation strategies. The ultimate goal is to support policymakers, regulators, and healthcare managers in strengthening patient safety culture at scale in complex health systems.

## Methods

### Study design and context

**Fig. 1:**
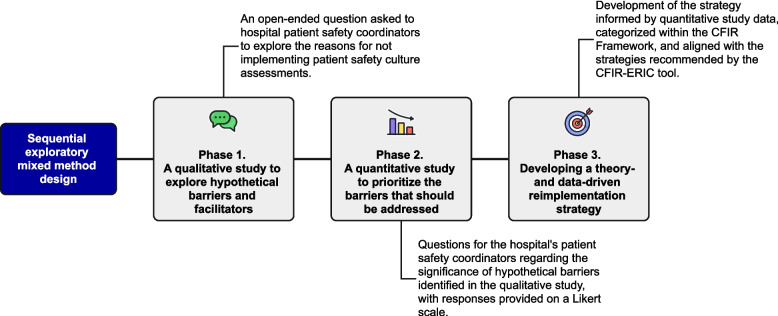
Sequential exploratory mixed-methods approach used to develop the implementation strategy

This is a sequential exploratory mixed-method study conducted in three phases (Fig. 1):

1. A qualitative study to identify barriers and facilitators to implementing PSC assessments, using an electronic survey with open-ended questions. Responses were classified according to the domains and constructs of the Updated CFIR [8].
2. A quantitative prioritization of the hypothetical barriers identified in Phase 1.
3. The design of an implementation strategy informed by the prioritized barriers, using the CFIR-ERIC Matching tool [10, 11] to align contextual determinants with implementation strategies.

The study was conducted between January and February 2024 and is embedded in national regulatory efforts to strengthen patient safety in Brazil. It supports the monitoring of goals established in the Integrated Plan for the Sanitary Management of Patient Safety in Health Services (2021–2025), coordinated by the Brazilian Health Regulatory Agency (Anvisa). This plan set a national target of having at least 40% of hospitals with intensive care units (ICUs) conducting PSC assessments by 2025 [4]. Despite two national campaigns (in 2021 and 2023), participation has remained low — around 15% [5, 6].

The intervention refers to the national implementation of the PSC assessment using the Brazilian electronic version of the Hospital Survey on Patient Safety Culture (HSOPS®), developed by the Agency for Healthcare Research and Quality (AHRQ) [17, 18].

The implementation strategy, which is the main object of this study, was designed to support the broader adoption of this assessment tool. Although developed in collaboration with Anvisa, the strategy reflects a scientific implementation design based on empirical findings. Its formal adoption as a national policy requires further deliberation by regulatory authorities.

The Brazilian hospital system includes both public hospitals (part of the Unified Health System—SUS) and private hospitals, which may provide care independently (e.g., via private insurance or out-of-pocket payments) or through contracts with SUS. All hospitals are legally required to promote a culture of patient safety and to establish Patient Safety Centers (NSPs). However, participation in the national PSC assessments remains voluntary. Organizational challenges—such as staffing limitations, leadership turnover, and competing institutional priorities—are common across hospital types and contributed to the barriers and strategic priorities identified in this study.

### Participants and recruitment

The target population included coordinators of Patient Safety Centers (Núcleos de Segurança do Paciente—NSPs) from public and private hospitals in Brazil with at least one ICU bed. The total number of eligible hospitals was 2,150, according to Anvisa’s national database. These professionals are responsible for promoting PSC assessments within their institutions and are key informants for understanding barriers to implementation.

Eligibility criteria included:

- Being an NSP coordinator in Anvisa’s database or in the databases of State Health Surveillance Units (Vigilância Sanitária Estadual, VISA).
- Having worked in an eligible hospital during 2023.
- Not having participated in the national PSC assessment in 2023 (since the focus was to understand non-adherence).

A census approach was used. Invitations were sent to all NSP coordinators registered in Anvisa’s official mailing list (*n* = 940) and disseminated through the VISA offices in each of Brazil’s 27 Federation Units.

### Phase 1. Qualitative study to identify barriers and facilitators

A qualitative survey was conducted using an electronic questionnaire in LimeSurvey. The questionnaire contained five structured questions designed to explore barriers and facilitators to the adoption of PSC assessments (Table 1). Participants were asked to provide open-ended responses based on their experiences.

**Table 1:** Questions included in the qualitative survey to explore barriers and facilitators to implementing PSC assessments

Question	Purpose
Did your hospital participate in the 2023 PSC assessment?	Screening question. Only those who answered “No” proceeded
What were the barriers that prevented participation?	To elicit barriers, later categorized using CFIR domains
If you were in charge, what would you do to increase hospital participation?	To elicit facilitators or suggested solutions
What is your role?	To confirm eligibility (only NSP coordinators could participate)
What is your state?	To collect contextual information (geographical location)

The first question was a screening item to verify whether the hospital had participated in the 2023 national PSC assessment. Only participants who answered “No” proceeded to the questions about barriers (Question 2) and suggestions to improve participation (Question 3). Question 4 confirmed the respondent’s role as an NSP coordinator, and Question 5 collected geographic information (state).

Participants were invited to describe barriers and facilitators freely.

The invitation to participate in the survey was sent on January 25, 2024, with a deadline of 10 days for respond. To maximize response rates, a multi-step strategy was applied: (1) an initial invitation sent by Anvisa; (2) a request for each State Health Surveillance Unit (NSP VISA) to forward the invitation to the NSP coordinators in their jurisdiction; and (3) a reminder email sent on the seventh day of the consultation period.

After data collection, we applied exclusion criteria to improve data quality: (1) respondents from hospitals that did not operate throughout 2023; (2) respondents who reported being unaware of whether a PSC assessment was conducted in their hospital in 2023; and (3) respondents who confused the PSC assessment with the National Assessment of Patient Safety Practices, which is a different assessment promoted by Anvisa.

Participants’ open-ended responses were categorized using the updated CFIR domains and constructs [8]. The coding process involved two independent researchers with formal training in patient safety and implementation science. One researcher performed the initial coding, and the second independently reviewed the classification. Disagreements were resolved through consensus meetings.

Participants’ statements about difficulties in implementation were classified as barriers, while statements suggesting potential improvements were classified as facilitators.

Both barriers and facilitators were categorized into one or more of the 46 constructs of the CFIR, grouped into five major domains:

- Innovation domain (8 constructs): characteristics the intervention itself (in this case, the PSC assessment).
- Outer setting domain (7 constructs): external factors influencing the implementation, such as regulatory demands of patient needs.
- Inner setting domain (11 constructs): organizational characteristics within the hospital, including culture, infrastructure, and resources.Individuals domain (9 constructs): characteristics, attitudes, and skills of the people involved in implementation.
- Implementation process domain (11 constructs): processes related to planning, engaging, executing, and reflecting on implementation activities.

These results generated a comprehensive set of hypothetical barriers and facilitators, which were then carried forward to the quantitative phase for prioritization.

### Phase 2. Quantitative study of the importance of the hypothetical barriers

The hypothetical barriers identified in the qualitative phase were converted into 23 items in a structured questionnaire administered through LimeSurvey. We refer to them as “hypothetical barrier” because they were derived from a qualitative study and had not yet been quantitatively validated as relevant barriers in the national context. This quantitative phase aimed to verify the perceived importance of each barrier from the perspective of patient safety coordinators.

For each item, the question was: “In your experience, was this barrier important to implementing the patient safety culture assessment?”. Responses were recorded on a five-point Likert scale: 1- Strongly Disagree; 2- Disagree; 3- Neutral; 4- Agree; 5- Strongly Agree.

The questionnaire was distributed to the same population as the qualitative phase on February 7, 2024, with a response window of 10 days. The same strategy used in Phase 1 to improve response rates was applied, including direct invitations from Anvisa, requests to State Health Surveillance Units (VISA) to forward the invitations, and a reminder sent on the seventh day. Data were collected using LimeSurvey.

Descriptive analysis was performed to calculate the frequency of agreement for each hypothetical barrier (i.e., the proportion of respondents who selected “Agree” or “Strongly Agree”). A Pareto diagram was constructed to prioritize the most relevant barriers contributing to the implementation gap.

This phase concluded with the synthesis of the most important barriers into a theoretical causal model of the problem, structured using the CFIR Framework.

### Phase 3. Design of the implementation strategy based on prioritized barriers

The objective of the third phase was to develop an implementation strategy based on the barriers prioritized in Phase 2. We followed the approach proposed by Waltz et al. (2019) [10], using the CFIR-ERIC Matching Tool (Version 0.5x). This tool is based on the collective judgment of 169 implementation science experts who identified and ranked up to seven strategies most likely to address each CFIR barrier. Implementation strategies were selected from the 73 strategies catalogued in the ERIC compilation [9, 10].

To inform the selection of strategies, we considered both expert consensus data and contextual fit. The CFIR-ERIC Matching Tool includes, for each CFIR construct, a list of implementation strategies and the corresponding “Recommended by Experts” (RBE) percentage. This proportion reflects the share of experts who endorsed each strategy as appropriate for addressing a given barrier. In our study, we used RBE values as a guiding reference to prioritize strategies with higher expert endorsement. However, no strict cutoff was applied; contextual relevance to the Brazilian hospital setting was also considered in the final selection.

A driver diagram was developed to visually organize the implementation strategy. The first column presents the general goal of reaching 40% of hospitals with PSC assessments by 2025. The second column identifies the four CFIR domains where barriers were located. The third column details the eight CFIR constructs within these domains that were prioritized on the Pareto analysis. The fourth column presents 16 proposed implementation strategies designed to address these barriers and support the national dissemination and implementation of PSC assessments. The design of the implementation strategy was guided by five principles:

1. It should be multifaceted.
2. It should be data-driven, based on barriers considered important.
3. It should incorporate strategies recommended by experts (ERIC).
4. It should be concise and feasible.
5. It should include responsibilities assigned to an external agent (Anvisa), considering the application of responsive regulatory interventions [19].

Although the CFIR-ERIC Matching Tool was developed based on the original CFIR version, we used the updated CFIR framework for classifying barriers. When necessary, we adapted the strategy selection process using expert consensus to address constructs not fully mapped in the original ERIC recommendations.

### Ethical and financing aspects

This study was reviewed and approved by the Research Ethics Committee of the Federal University of Rio Grande do Norte—Hospital Universitário Onofre Lopes (approval number 5.501.454). The specific analysis presented in this manuscript was funded by the United Nations Development Program (UNDP). A Large Language Model (LLM), specifically ChatGPT (developed by OpenAI), was used to assist the authors in improving the clarity of the manuscript in English and reducing the word count. All content was generated under the authors’ direction, reviewed, and validated to ensure accuracy and appropriateness.

## Results

**Fig. 2:**
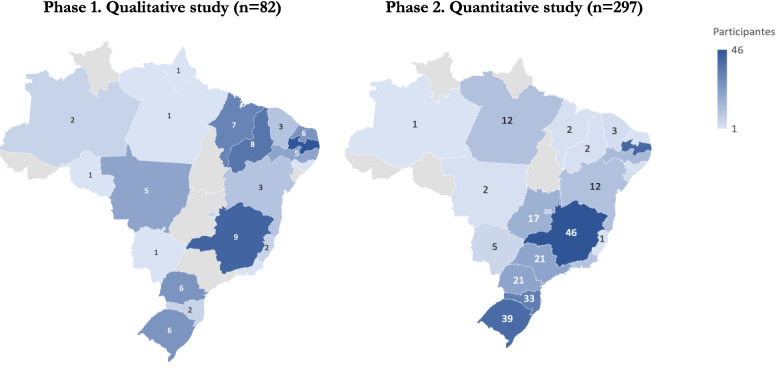
Geographic distribution of participants in the mixed-methods study

This study includes data from participants located across all five Brazilian regions (North, Northeast, South, Southeast, and Central-West) in both the qualitative and quantitative phases. Hospitals from 21 of the 27 federation units were represented in the qualitative phase (*n* = 82), and 23 of 27 in the quantitative phase (*n* = 297), ensuring broad geographic diversity (Fig. 2).

Initial invitations were sent to 940 NSP coordinators. Attrition in the qualitative phase occurred due to ineligibility (e.g., respondents working in hospitals that were inactive in 2023, uncertain about assessment participation, or confusing the PSC assessment with another national tool). These exclusion criteria are detailed in the Methods section. The final sample represents a meaningful cross-section of Brazilian hospitals with ICUs, both public and private.

### Hypothetical barriers and facilitators identified in the qualitative study

Open-ended responses from participants were classified across the five domains of the CFIR: Innovation, Outer Setting, Inner Setting, Characteristics of Individuals, and Implementation Process. A detailed database of all participant responses, along with their categorization into CFIR domains and constructs, is available in Supplement Material 1.

The classification of barriers and facilitators by CFIR domain is summarized in Table 2.

**Table 2:** Representative quotes from each CFIR domain and construct identified in participant responses

CFIR Domain and Construct^a^	Nº of Quotes	Example Quote
I. Innovation Domain (ID)		
Relative Advantage (C)	9 B, 2 F	“The institution has another methodology of its own to assess the perception of safety culture.” – RS
Adaptability (D)	1 B, 1 F	“Access to the questionnaire only through an electronic form makes it very difficult to access in all areas of the hospital…” – RS
Complexity (F)	9 B, 22 F	“The team says the questionnaire is too long.” – RN
Design (G)	6 B, 7 F	“Registering professional respondents is not easy.” – MA
II. Outer Setting Domain (OSD)		
Policies & Laws (E)	1 B, 4 F	“I would make it mandatory for hospital management and the NSP to carry out a safety culture assessment” – AM
III. Inner Setting Domain (ISD)		
Structural Characteristics (A) – IT Infrastructure	7 B	“[We don’t have] the employees’ email address.” – MT
Structural Characteristics (A)—Work Infrastructure	19 B, 3 F	“[We were] restructuring the Patient Safety Center.” – BA
Culture (D)	1 B, 1 F	“We need to strengthen our culture and protocols.” – RN
Culture (D)—4. Learning-Centeredness	1 B	“Professionals don’t adhere to the survey; they don’t think it’s important.” – RN
Relative Priority (G)	6 B	“Due to some internal demands, I was unable to administer the survey this year.” – PB
Available Resources (J)—3. Materials & Equipment	1 B	“Another problem is that the internet does not work throughout the hospital…” – RN
Access to Knowledge & Information (K)	2 B, 2 F	“I think it would be important to have more clarification about the survey….” – PE
IV. Individuals Domain (IndD)		
High-level leaders (A)	5 B	“New management and many new employees who do not understand and have not undergone any training…” – PI
Mid-level leaders (B)	1 B	“Incipient support for health professionals and hospital sector coordinators.” – RN
Implementation Leads (E)	2 B	“I couldn’t understand how to enter the system to carry out the evaluation…” – RO
Innovation Delivers (H)	1 F	“The manager must formalize the request for completion in a mandatory manner… – MT
Innovation Recipients (I)	7 B	“Resistance and disruptive behavior” – MT
V. Implementation Process Domain (IPD)		
Engaging (F)	6 B, 14 F	“I did not receive any emails about this assessment, so I did not participate.” – MA
Reflecting & Evaluating (H)	1 F	“Show assessment results more clearly after survey completion.” – PE

Federation unit codes represent the state of the participant who provided the quote. Only constructs mentioned by participants are included. A full list of quotes is available in Supplementary File 1

*B* Barrier, *F* Facilitator

^a^Constructs are presented according to the Updated CFIR Framework

- In the Innovation domain, participants most frequently reported issues related to the constructs “Innovation Complexity” (31 quotes: 9 barriers and 22 facilitators) and “Innovation Design” (13 quotes: 6 barriers and 7 facilitators). For example, a patient safety coordinator from a hospital in Rio Grande do Norte (RN) commented on a barrier related to complexity: “The team says that the questionnaire is too long”. Statements about innovation design referred to technical difficulties, such as registering employees’ emails in the electronic system.
- In the Outer Setting domain, barriers and facilitators were mainly linked to the construct “Policies and Laws” (5 quotes: 1 barrier, 4 facilitators). This reflects difficulties in engaging professionals in a non-mandatory activity. For instance, a participant from a hospital in Amazonas (AM) suggested: “I would make it mandatory for hospital management and the NSP to carry out a safety culture assessment”.
- In the Inner Setting domain, two constructs were predominant: “Structural Characteristics” (29 quotes — 7 related to information technology infrastructure [7 barriers] and 22 related to work infrastructure [19 barriers, 3 facilitators]) and “Relative Priority” (6 quotes: 6 barriers). Barriers in structural characteristics involved lack of access the professionals’ emails and NSP organizational challenges. As an example, a participant from Bahia (BA) stated: “[We were] restructuring the Patient Safety Center.” Quotes classified under “Relative Priority” revealed that the PSC assessment was deprioritized in favor accreditation and mandatory training demands.
- In the Characteristics of Individuals domain, barriers were concentrated in “High-level leadership” (5 quotes: 5 barriers). Barriers related to leadership refer to lack of motivation or support for the PSC assessment. Barriers among “Innovation Recipients” (7 quote: 7 barriers) included low motivation to complete the survey and difficulties using electronic platforms. A participant from Mato Grosso (MT) noted: “Resistance and disruptive behavior” as a barrier affecting adoption.
- In the Implementation Process domain, the construct “Engaging” captured most of the barriers and facilitators (20 quotes: 6 barriers, 14 facilitators). Participants frequently reported a lack of information and formal invitation to participate in the PSC assessment. As one participant from Maranhão (MA) stated: “I did not receive any emails about this assessment, so I did not participate.”.

### Perceived importance of hypothetical barriers in the quantitative study

After qualitatively identifying the hypothesized barriers and facilitators, we assessed their perceived importance from the perspective of implementers. NSP coordinators were asked to rate the relevance of 23 hypothetical barriers identified in the qualitative phase (Table 3). The frequency of agreement (“Agree” and “Strongly Agree”) ranged from 15.5% to 73.4%.

**Table 3:** Frequency distribution of agreement levels on hypothetical barriers to implementing PSC assessments, based on responses from 297 patient safety coordinators

Survey Items Grouped by CFIR Domains	Disagree/Strongly disagree	Neutral	Agree/Strongly agree
	**n (%)**	**n (%)**	**n (%)**
I. Innovation Domain (ID)			
ID 1. There are other tools that are better than the E-Questionnaire to assess patient safety culture.	138 (46.5)	76 (25.6)	83 (27.9)
ID 2. It is difficult to register participants in the electronic questionnaire.	160 (53.9)	33 (11.1)	104 (35.0)
ID 3. The survey used is too long with too many questions.	96 (32.3)	22 (7.4)	179 (60.3)
ID 4. The questions in the questionnaire are complex.	154 (51.9)	40 (13.5)	103 (34.7)
ID 5. The electronic questionnaire platform is confusing and difficult to understand.	180 (60.6)	45 (15.2)	72 (24.2)
ID 6. The App for face-to-face data collection only works on Android and should also work on IOS.	30 (10.1)	104 (35.0)	163 (54.9)
ID 7. The platform crashed and I was unable to use it.	139 (46.8)	66 (22.2)	92 (31.0)
II. Outer Setting Domain (OSD)			
OSD 1. The national assessment of patient safety culture should be mandatory.	73 (24.6)	25 (8.4)	199 (67.0)
III. Inner Setting Domain (ISD)			
ISD 1. We do not have enough resources (computers, tablets) to administer electronic surveys.	138 (46.5)	14 (4.7)	145 (48.8)
ISD 2. The hospital does not have easy access to professional’s emails.	133 (44.8)	20 (6.7)	144 (48.5)
ISD 3. The Patient Safety Center is not well organized.	224 (75.4)	27 (9.1)	46 (15.5)
ISD 4. PSC assessment is not considered important in my institution.	232 (78.1)	18 (6.1)	47 (15.8)
ISD 5. The Patient Safety Center is always overloaded with activities.	93 (31.3)	29 (9.8)	175 (58.9)
ISD 6. Patient safety actions are not valued in my hospital.	187 (63.0)	23 (7.7)	87 (29.3)
ISD 7. There is a lack of training on how to assess patient safety culture.	76 (25.6)	17 (5.7)	204 (68.7)
IV. Individuals Domain (IndD)			
IndD 1. Top management does not get involved or demand PSC assessment.	157 (52.9)	33 (11.1)	107 (36.0)
IndD 2. Sector coordinators do not help promote the questionnaire.	131 (44.1)	38 (12.8)	128 (43.1)
IndD 3. Professionals are resistant to responding to the PSC survey.	75 (25.3)	40 (13.5)	182 (61.3)
V. Implementation Process Domain (IPD)			
IPD 1. The national PSC assessment is not widely disseminated.	67 (22.6)	12 (4.0)	218 (73.4)
IPD 2. Health surveillance and SUS do not emphasize PSC assessment.	136 (45.8)	34 (11.4)	127 (42.8)
IPD 3. Support from state health surveillance has been insufficient.	143 (48.1)	60 (20.2)	94 (31.6)
IPD 4. Support from the university managing the platform is insufficient.	87 (29.3)	134 (45.1)	76 (25.6)
IPD 5. The national PSC assessment should be communicated earlier.	62 (20.9)	35 (11.8)	200 (67.3)

The statements were rated on a 5-point Likert scale: 1 = Strongly disagree, 2 = Disagree, 3 = Neutral, 4 = Agree, 5 = Strongly agree. Responses were grouped into three categories for analysis

*PSC* Patient Safety Culture

**Fig. 3:**
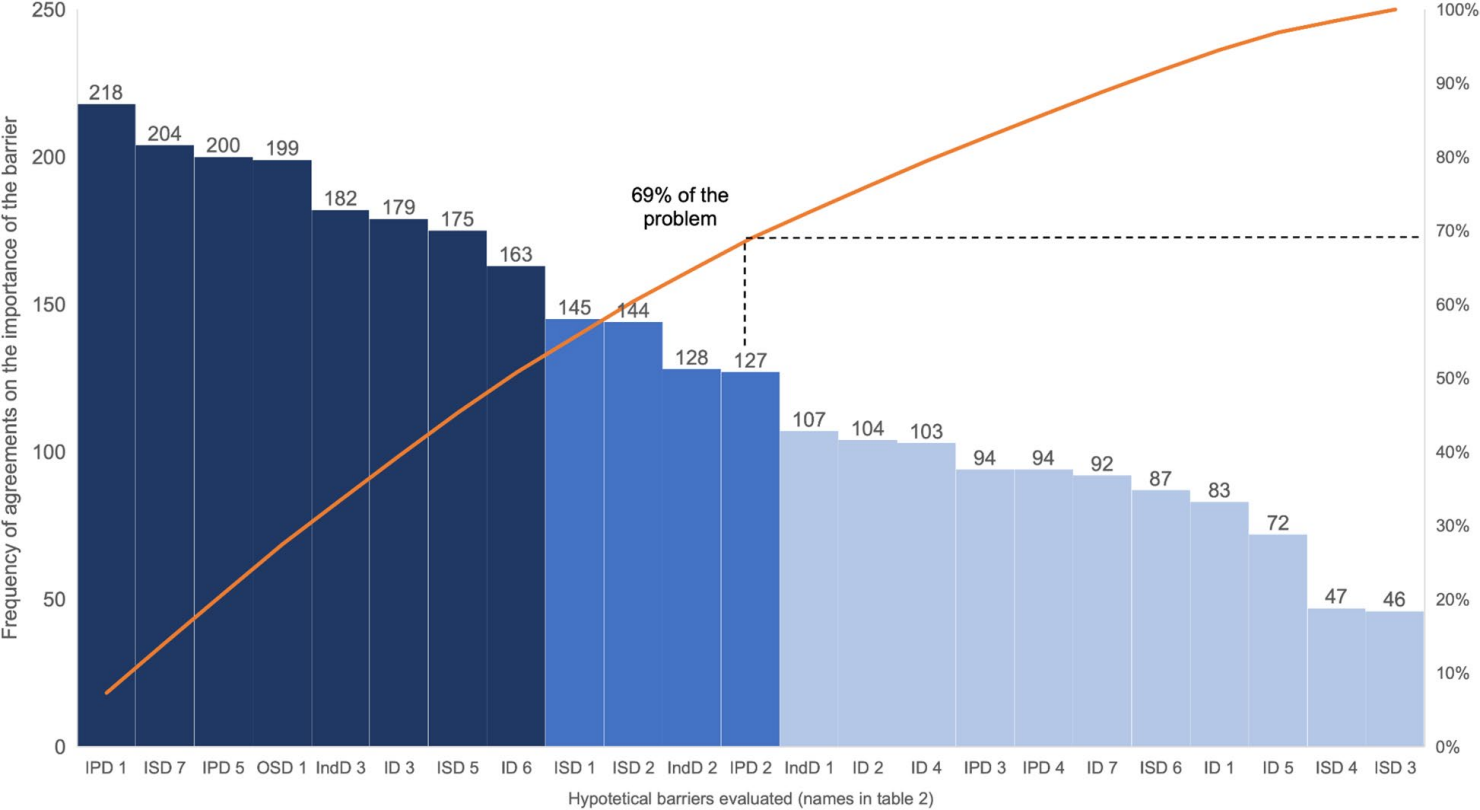
Pareto diagram of hypothetical barriers’ importance, analyzed by 297 patient safety center coordinators

The Pareto chart (Fig. 3) organizes the hypothetical barriers in descending order of Absolute Frequency (AF), which represents the sum of “Agree” and “Strongly Agree” responses. This was used to prioritize which barriers should be addressed in the implementation strategy design.

While Table 3 and Fig. 3 already summarize the most relevant barriers by frequency and CFIR domain, we also present below the top-ranked individual barriers with their absolute frequencies to enhance clarity and transparency, as suggested by a reviewer.

The most relevant barriers, in order of importance, were:

1. The national PSC assessment is not widely disseminated — Implementation Process domain (AF = 218);
2. There is a lack of training on how to conduct the PSC assessment — Inner Setting domain (AF = 204);
3. The PSC assessment should be communicated earlier to hospitals — Implementation Process domain (AF = 200);
4. The PSC assessment should be mandatory — Outer Setting domain (AF = 199);
5. Professionals are resistant to completing the PSC survey — Characteristics of Individuals domain (AF = 182);
6. The PSC survey is too long, with too many questions — Innovation domain (AF = 179)
7. Patient Safety Centers are overloaded with excessive tasks relative to their staffing capacity — Inner Setting domain (AF = 178).
8. The app for face-to-face data collection works only on Android and should be available for IOS as well — Innovation domain (AF = 170).

These eight barriers account for 51% of the total cumulative frequency of agreements responses, representing the most significant contributors to the difficulty in implementing PSC assessments. Expanding the analysis to the top 12 barriers increases the cumulative contribution to 69%, while the remaining 11 barriers account for 31%.

**Fig. 4:**
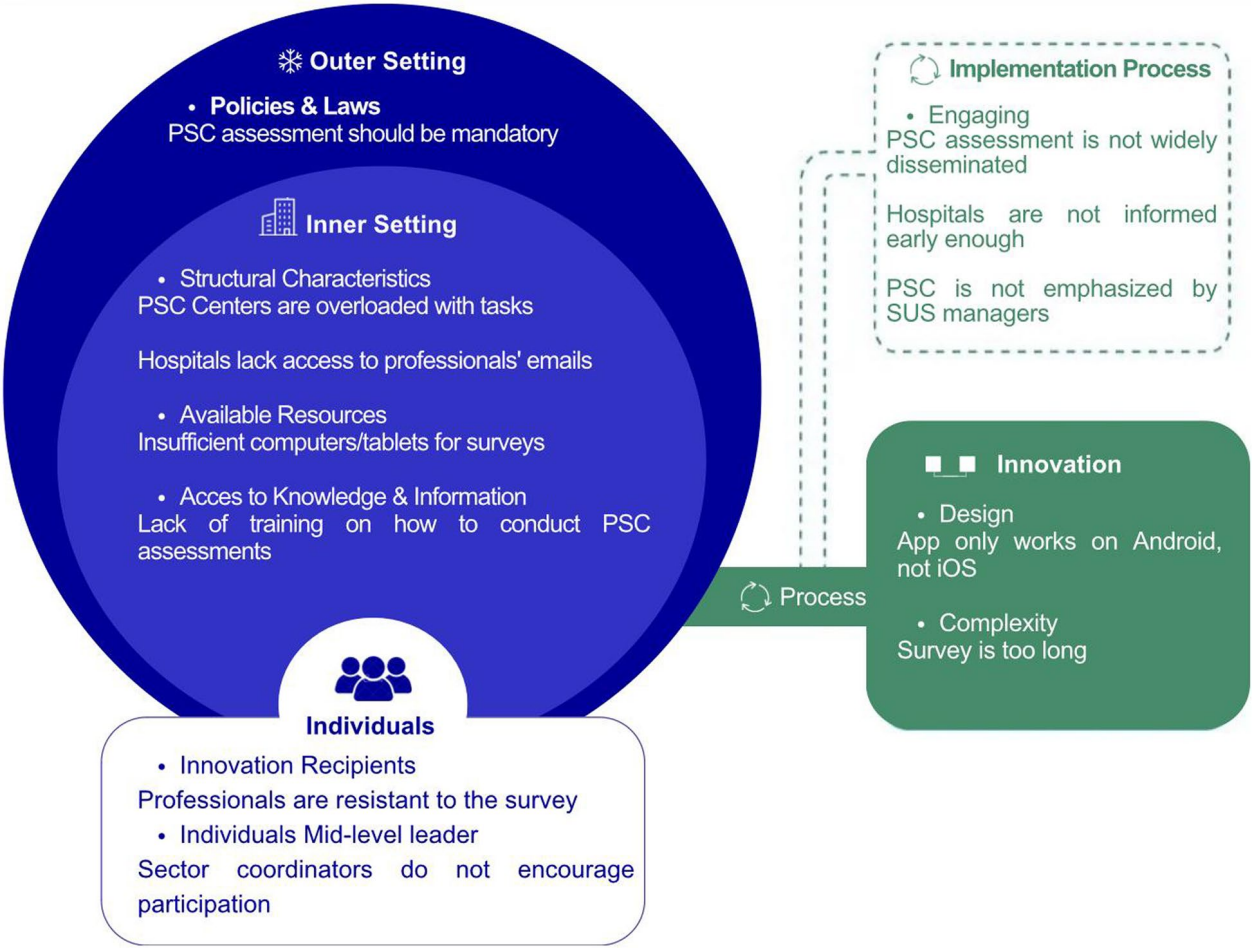
Key barriers to PSCA uptake in Brazil mapped to CFIR domains and constructs, adapted from CFIR

The most relevant barriers identified in the quantitative phase were synthesized using the Updated CFIR framework to support the design of the implementation strategy. Figure 4 presents a visual map of these barriers, organized by CFIR domain and construct. Each domain—*Innovation*, *Outer Setting*, *Inner Setting*, *Characteristics of Individuals*, and *Implementation Process*—is represented with its respective prioritized constructs and associated barriers, based on frequency of agreement in the national survey. To enhance clarity, the barriers are expressed in concise statements. Rather than presenting a causal chain, the figure offers a structured representation of how technical, organizational, individual, contextual, and procedural challenges are distributed across the CFIR domains. This framework-informed synthesis provided the conceptual foundation for selecting targeted strategies to improve the uptake of patient safety culture assessments.

### Implementation strategy designed with support of the CFIR-ERIC tool

**Fig. 5:**
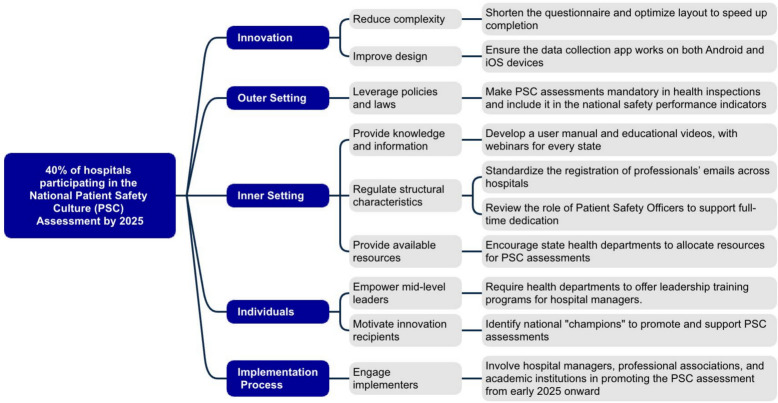
Proposed model to enhance the implementation of Patient Safety Culture Assessments (PSCA) in Brazil

The implementation strategy (phase 3) was developed based on the prioritized barriers identified in the quantitative phase. This strategy is visually represented in a driver diagram (Fig. 5), which organizes its components into four columns: (1) the general goal, (2) the CFIR’s domains, (3) the CFIR constructs, and (4) the implementation strategies to address each barrier.

The general goal of the strategy is to achieve 40% of hospitals conducting the PSC assessment by 2025. The barriers are distributed within four CFIR domains, mapped onto the eight constructs identified as priorities. The strategy proposes twelve implementation strategies aimed at reducing barriers and facilitating national adoption of the PSC assessment. Each implementation strategy is linked to a corresponding ERIC classification.

In addition to what is visually represented in Fig. 5, the selection of implementation strategies was informed by the Recommended by Experts (RBE) percentages from the CFIR-ERIC Barrier Buster Tool (Version 0.53). These percentages reflect the proportion of 169 implementation science experts who endorsed each strategy as appropriate for addressing a given CFIR barrier.

Examples of the selected implementation strategies include: altering incentive structures for the Policy and Laws construct (41% RBE); changing physical structure and equipment to improve Available Resources (48% RBE); and conducting education meetings (79% RBE), developing educational materials (59% RBE) — all associated with the construct “Access to Knowledge and Information”. Additional strategies include identifying and preparing champions for “Opinion Leaders” (64% RBE); promoting adaptability to address “Innovation Complexity” (73% RBE); and revising professional roles for “Structural Characteristics” (18% RBE).

The implementation strategy was designed to be multifaceted, addressing multiple barriers simultaneously; data-driven, grounded in barriers prioritized by the implementers themselves; evidence-informed, incorporating strategies endorsed by the CFIR-ERIC expert consensus; and feasible, considering operational constraints. Additionally, the strategy is intended to be supported by an external agent (Anvisa), including the application of responsive regulatory interventions to ensure scalability, sustainability, and national reach.

## Discussion

This study highlights a multifaceted, data-driven and feasible strategy — supported by a responsive regulatory approach — to address the persistently low uptake of PSC assessments in Brazilian hospitals. Using the Updated CFIR [8], we identified and quantified key barriers to implementation, and designed a theoretically grounded, context-specific reimplementation strategy based on the CFIR-ERIC framework.

To our knowledge, this is the first prospective application of both the Updated CFIR and the CFIR-ERIC framework in designing a national-level implementation strategy for PSC assessments in a middle-income country. The proposed strategy builds on the WHO’s Global Action Plan for Patient Safety 2021–2030 [1], which recommends periodic culture assessments in all member states’ health systems.

Despite a growing international consensus on the importance of assessing patient safety culture (PSC), including its inclusion as a global indicator in Strategic Objective 2 of the WHO Global Patient Safety Action Plan 2021–2030, most of the international literature remains focused on other aspects. These include comparative analyses of PSC survey results across countries and institutions [20, 21], psychometric evaluations of measurement tools [22], investigations of the relationship between safety culture and adverse events [23], and strategies to improve culture within individual health services [24]. Several countries, including the United States, Brazil, and Portugal, have carried out national initiatives to assess PSC using versions of the Hospital Survey on Patient Safety Culture (HSOPS). In the United States, these efforts were supported by the Agency for Healthcare Research and Quality (AHRQ) and federal programs such as the Comprehensive Unit-based Safety Program (CUSP), with widespread dissemination and periodic national reports. In Brazil, national evaluations were conducted in 2021 and 2023, led by the national regulatory agency (Anvisa), and Portugal has supported multicenter assessments using HSOPS as well. However, even in these contexts, we did not find published studies that describe structured, theory-based implementation strategies specifically designed to promote the uptake of PSC assessments on a national scale. This study helps fill that gap by proposing a multifaceted, empirically grounded implementation strategy to support broader adoption of PSC assessments in Brazil, guided by the CFIR and ERIC frameworks.

This study included respondents from all regions of the country, which increases the possibility of generalization regarding findings about barriers and facilitators. Furthermore, we were careful not to design the national strategy to resolve the problem based solely on the qualitative study. Complementing the barrier analysis with the quantitative study allowed the hypothetical barriers identified in the qualitative study to be validated, at least from the perspective of the national sample of those responsible for hospital patient safety. This step-by-step exploratory mixed method follows the phases of Saturno’s [25] quality improvement cycles, whereby he recommends analyzing the causes of the problem followed by a quantitative study of the hypothetical causes, before designing improvement interventions.

Progressing the implementation of patient safety culture assessments will require a multi-domain approach. The Brazilian health system has aligned with global demands for periodic PSC assessments [4], but adherence remains challenging [5, 6]. ICU hospital participation increased from 271 to 320 between 2021 and 2023, yet the relative frequency stayed at 15% as ICU hospitals grew from 1,856 to 2,150 post-pandemic. The expanding target population added complexity, and this study identified barriers across nine constructs within five CFIR domains.

Barriers related to innovation stem from its complexity and design. The PSC assessments rely on an online platform that facilitates survey administration, sends automated reminders, and analyzes results. However, challenges include the questionnaire’s length and software usability. While electronic tools improve efficiency, they must also be user-friendly and concise [26]. The study proposes adopting the Hospital Survey on Patient Safety Culture 2.0, which has 10 fewer items than the original, maintains strong psychometric properties, and is faster to complete [27]. Though tested in Brazil, it is still being integrated into Anvisa’s electronic system [28].

The Inner Setting and Individuals domains highlight organizational barriers. Patient safety officers face excessive workloads, lack standardized communication tools (e.g., professional email lists), and encounter motivational challenges among leaders and staff. These issues reflect a low safety and learning culture in Brazilian hospitals [6], as also reported in other low- and middle-income countries [20]. Weak internal commitment to safety assessments impacts full-time staffing, communication effectiveness, and participation in safety initiatives. The study suggests exclusive safety officer roles and “champions” to engage leaders and raise awareness — strategies aligned with prior experiences in the U.S. Veterans Health Administration and Japan [14, 15].

In the Outer Setting and Implementation Process domains, hospitals reported insufficient external demands for assessments and poor communication from Anvisa. Contributing factors include outdated contact registers, staff turnover, and limited engagement by SUS (Sistema Único de Saúde; Unified Health System in English) management. Similar challenges were also reported in other national-scale studies, such as in Guatemala [13]. Improved communication strategies and stronger involvement from professional societies and managers are essential to address these gaps.

These findings and the proposed strategy contribute directly to advancing the WHO’s Global Patient Safety Action Plan 2021–2030 [1]. Specifically, they align with Strategic Objective 2, which promotes the development of high-reliability health systems. One of the core indicators for this objective is the regular implementation of PSC assessments in healthcare settings. By addressing key contextual barriers and proposing actionable strategies for large-scale implementation, our study supports both the national goals of Anvisa and the broader global commitment to system-level safety improvement.

This study’s findings informed a strategy targeting 69% of identified barriers, adhering to principles of intervention feasibility and efficiency given regulatory resource constraints. Specific actions to address access to knowledge and information are already underway. A user manual and six instructional videos were developed and published on the Anvisa website to support Brazilian health services [29]. These resources will be widely disseminated for the 2025 evaluation.

This study represents the first use of implementation science frameworks to accelerate PSC assessment implementation through regulatory actions within Anvisa. Despite its promise, the study has limitations: (1) The qualitative approach included responses from 72 Brazilian hospitals but does not capture all possible barriers. Generalizations about the perceived importance of barriers should be made cautiously. Nonetheless, the results provide a valuable approximation of reality and support decision-making in a large, dispersed hospital population like Brazil’s. (2) The CFIR-ERIC tool [10, 11] guided intervention selection but had limitations. It is based on the original CFIR, while we used the updated version, complicating intervention alignment for some constructs. Additionally, as a generic tool, many proposed actions are more applicable when the implementation designer works within health services, which was not our profile as policymakers and regulators. Thus, expert-recommended ERIC strategies were not always ideal for our national intervention goal. For regulatory interventions, other macro-level quality improvement frameworks may be more suitable [19, 30, 31]. (3) The study did not consider NSP VISA’s perspective within SNVS on barriers and facilitators for PSC assessment, which future studies should address, as they are also key implementers.

In addition to the limitations mentioned, this study relied on self-reported data, which may introduce response bias. Participation was voluntary, raising the possibility of selection bias, and the results were not triangulated with observational or performance data. Finally, while the CFIR-ERIC Tool provided structured guidance, it was not fully aligned with the updated CFIR framework, requiring interpretive adaptations.

## Conclusions

Multiple barriers hinder the implementation of PSC assessments in Brazilian hospitals, including challenges in organizing safety centers, limitations in information systems for distributing survey invitations, and insufficient knowledge about PSC evaluation methods. Implementers at the service level also face a lack of incentives or regulatory requirements to participate in national safety initiatives, as well as resistance from some organizational members. A more assertive and proactive dissemination strategy is needed to increase engagement. The implementation strategy designed in this study is multifaceted, data-driven, concise, and grounded in principles of responsive regulation. Its impact will be assessed in 2026, following the nationwide PSC assessment planned by Anvisa for 2025.

## Supplementary Information

Supplementary Material 1.

## Data Availability

The datasets generated and/or analyzed during the current study are available from the corresponding author upon reasonable request. No identifiable or sensitive information is included in the dataset.

## Abbreviations

WHO: World Health Organization
PSC: Patient Safety Culture
PSC assessments: Patient Safety Culture assessments
ICUs: Intensive care units
SNVS: National Health Surveillance System (Sistema Nacional de Vigilância Sanitária)
CFIR: Consolidated Framework for Implementation Research
ERIC: Expert Recommendations for Implementation Change
Anvisa: Brazilian Health Regulatory Agency
SOPS®: Surveys on Patient Safety Culture (by AHRQ)
AHRQ: Agency for Healthcare Research and Quality
NSP: Patient Safety Centers (Núcleos de Segurança do Paciente)
ID: Innovation domain
OSD: Outer Setting domain
ISD: Inner Setting domain
IndD: Characteristics of Individuals domain
IPD: Implementation Process domain
RBE: Recommended By Experts
UNDP: United Nations Development Program
RN: Rio Grande do Norte
AM: Amazonas
BA: Bahia
MT: Mato Grosso
MA: Maranhão
AF: Absolute Frequency
