# Supplementary material for: Enhancing adoption of patient safety culture assessments in Brazil: a strategy informed by CFIR and ERIC
Source: Implement Sci Commun. 2026 Jan 17;7:31. doi: 10.1186/s43058-026-00865-7 (PMC12895655; doi:10.1186/s43058-026-00865-7)
Supplement: Supplementary file 1 — Supplementary Material 1. [file 43058_2026_865_MOESM1_ESM.pdf]

# Appendix A.

## Participants statements classified by the Updated Consolidated Framework for Implementation Research – (CFIR).

In each construct of the following tables, the statements that represent barriers are numbered and identified by B1, B2, B3, etc. Likewise, the statements that represent facilitators are numbered and identified by F1, F2, F3, etc.

| **I. Innovation domain:** The "thing" being implemented, that is "the patient safety culture assessments". | | |
|---|---|---|
| **Construct name** | **Participant's statements classified by the CFIR construct** | |
| A. Innovation Source | None. | |
| B. Innovation Evidence Base | None. | |
| C. Innovation Relative Advantage | B1 | "The hospital where I work already carries out another internal survey of the patient safety culture." – PI |
| | B2 | "Yes, we have an annual schedule of activities, always approved in December of the previous year. In the case of this assessment, we have already agreed with [our accreditation councils] to administer the survey every six months, so we are no longer able to adhere to this national evaluation, as it becomes unfeasible in our agenda." – PR |
| | B3 | "The institution has another methodology of its own to assess the perception of safety culture." – RS |
| | B4 | "We have our own E-Questionnaire at the institution." – PA |
| | B5 | "The same survey had already been carried out in a period similar to the proposed by health surveillance." – PR |
| | B6 | "We chose another internal assessment tool." – PE |
| | B7 | "We already conducted an annual AHRQ-based survey with 50 questions. We believe that it would be unfeasible to apply a similar new one." – DF |
| | B8 | "We administered the hospital survey on patient safety culture version 02 in partnership with the University of Florida practically in the same period." – MG |
| | B9 | "We had already carried out the survey using another questionnaire." – CE |
| | F1 | "Send proof that the hospital carried out the survey using its another E-Questionnaire" – PA |
| | F2 | "Get the results from hospitals that have already carried out the survey, so we don't have to administer two surveys a year." – DF |
| D. Innovation Adaptability | B1 | "Access to the questionnaire only through an electronic form makes it very difficult to access in all areas of the hospital, due to the need for a computer, smartphone or tablet." – RS |
| | F1 | "Also provide a questionnaire in printed format for employees to fill out." – RS |

| | | |
|---|---|---|
| E. Innovation Trialability | None. | |
| F. Innovation Complexity | B1 | “Resistance due to the questionnaire being too long” – PB |
| | B2 | “Very extensive”. – PB |
| | B3 | “Very extensive questionnaire. The team reported that it took a long time and was confusing” – MG |
| | B4 | “Large questionnaire, very broad, time– consuming to answer. I realized it was difficult for employees to understand.” – RS |
| | B5 | “Long questionnaire”. – PI |
| | B6 | “The size of the questionnaire makes it difficult for professionals to adhere.” – PI |
| | B7 | “Extensive electronic questionnaire, low adherence in 2021 and 2022, with many difficulties for professionals to adhere.” – PI |
| | B8 | “The team says the questionnaire is too long.” – RN |
| | B9 | “Very long questionnaire” – RN |
| | F1 | “Reduce questionnaire items” – PE |
| | F2 | “Making the questionnaire simpler and faster.” – MG |
| | F3 | “If you can, make the instrument more concise.” – MT |
| | F4 | “Use short and objective questionnaires.” – PR |
| | F5 | “Reduce the number of questions.” – PB |
| | F6 | “The survey could be easier and simpler.” – RO |
| | F7 | “Reduce the number of questions” – PR |
| | F8 | “Use a simpler questionnaire, with fewer questions. Make the presentation of evaluation results clearer.” – MG |
| | F9 | “I would simplify the questionnaire as much as possible. I would think about the possibility of validating a tool with open questions only for health care and other general questions for all areas of the institution.” – RS |
| | F10 | “Facilitate the registration of professionals in the system.” – MA |
| | F11 | "I would put something simpler so that people have an easier time answering. There are a lot of questions." – PB |
| | F12 | “I would make the questionnaire shorter and ask fewer questions about the organizational climate in the survey.” – MG |
| | F13 | “I would make the questionnaire smaller” – MA |
| | F14 | “Make the questionnaire simpler. For example, the first step is divided into many parts, and they give up answering.” – AL |
| | F15 | “I would reduce the number of questions and try to associate questions that have similar answers.” – PI |
| | F16 | “Simplify: Reducing or better wording questions” – PI |
| | F17 | “I would reduce the number of questions, leaving only the most objective ones.” – MG |
| | F18 | “Reduce the number of questions” – PI |
| | F19 | “Make the questionnaire more accessible” – MA |

| | |
|---|---|
| | F20 “The need to collect professionals’ emails is a problem due to the lack of data. The size of the questionnaire is also scary, they complain mainly that it is not aimed at people in the sector.” Nurse<br>F21 “If I could, I would make the instrument shorter.” – RN<br>F22 "Easiest and most quickly accessible form for everyone" – PI |
| G. Innovation Design | B1 “The compliance sector did not approve the sending of information from the institution’s employees. He considered that the system does not have a data privacy policy.” –DF<br>B2 “Registering professional respondents is not easy.” – MA<br>B3 “Difficulty registering employees.” – MS<br>B4 “The digital platform is confusing and very “polluted”, even though it contains explanatory videos.” – RS<br>B5 "I was unable to access the system. I received a website unavailable message." –MG<br>B6 "We were unable to register professionals." – PI |
| | F1 “No need to register employees by email” – MT<br>F2 “The assessment could be applied in parts. Most employees are overworked, don’t pay attention, and don’t focus on understanding the issues.” – DF<br>F3 “I would increase the deadline for sending and make a link that could be sent by other means without the need for email.” – SC<br>F4 “Providing a QR code would make everyone’s life easier, because here in our institution we have the habit of using QR Codes.” – PR<br>F5 “I didn’t see any difficulties with the instrument when applying it. I think that when interpreting the data it is more confusing and it would be good to have a video explaining each of the answers, to facilitate understanding, but UFRN was super helpful answering our questions. That would be my only suggestion, perhaps it would be good to record a video with the instrument's translator. But be objective, showing how to interpret the answers." – RS<br>F6 “The App needs to be available on all platforms, both Android and IOS.” – MA<br>F7 “That access to the questionnaire was not just linked to email. May there be another way to make this questionnaire available.” – PI |
| H. Innovation Cost | None. |

| **II. Outer Setting domain:** The setting in which the Inner Setting exists, that is all context outside the hospital. | |
|---|---|
| **Construct name** | **Participant’s statements classified by the CFIR construct** |
| A. Critical Incidents | None. |
| B. Local Attitudes | None. |
| C. Local Conditions | None. |
| D. Partnerships & Connections | None. |

| | |
|---|---|
| E. Policies & Laws | B1 “Nursing staff change due to new bill.” – RN |
| | F1 "I would make it mandatory and punishable for those who don't respond." – PB |
| | F2 “I suggest making assessment mandatory and adopting an easy-to-apply questionnaire” – MG |
| | F3 “I would make answering the questionnaire mandatory for performance evaluation” – RN |
| | F4 “I would make it mandatory for hospital management and the NSP to carry out a safety culture assessment” – AM |
| F. Financing | None. |
| G. External Pressure | None. |

| **III. Inner Setting domain**: innovation is implemented (hospital, unit and teams). | |
|---|---|
| **Construct name** | **Participant’s statements classified by the CFIR construct** |
| A. Structural characteristics | The statements were classified into subconstructs 2 and 3. |
| 1. Physical Infrastructure | None. |
| 2. Information Technology Infrastructure | B1 “[We don’t have easy] access to the emails of hospital professionals.” – MG |
| | B2 “Not all employees have an email address, which makes accessing the survey difficult.” – PI |
| | B3 “Create a list of professionals’ contacts to enter into the system.” – PB |
| | B4 "[We don't have] the employees' email address." – MT |
| | B5 “What made it difficult to carry out the survey was the fact that employees needed to have an individual email address.” – PR |
| | B6 “Employees do not have an institutional email address, so we were unable to send the questionnaire to be answered.” – SC |
| | B7 “We had difficulty registering all the professionals’ emails, as there is no updated database at the hospital.” – MG |
| 3. Work Infrastructure | B1 “There was a change in NSP coordinators.” – AM |
| | B2 "NSP was being restructured." – AP |
| | B3 "We were restructuring the NSP." – MT |
| | B4 "The people at NSP changed." – ES |
| | B5 “The NSP has only two people and the demands were many. We didn’t make it, but we already have a schedule for 2024.” – PB |
| | B6 “There was a huge shortage of professionals, causing the team to be overloaded and prioritize patient care.” – MG |

| | |
|---|---|
| | B7 “The NSP had not been created” – AM |
| | B8 “I had difficulties because I was alone at NSP. I also confess that I didn't understand how I would do this, as we had a lot of staff layoffs. The year was very unstable.” – MT |
| | B9 “We were still planning how to do this while respecting employee buy-in.” – BA |
| | B10"[We were] restructuring the NSP." – BA |
| | B11“At the beginning of the year there was a change of government that led to changes in senior management. There was also a change of employees, with the arrival of many without training, especially in nursing, and in October the institution was moved to another building." – PI |
| | B12“The hospital was in the process of restructuring.” – PR |
| | B13“[We were] with the NSP in the implementation phase” – CE |
| | B14"The hospital has undergone administrative transitions." – PE |
| | B15“Difficulty in administering the survey due to the lack of administrative/IT support at the NSP.” –PB |
| | B16"The NSP was still being implemented." – MA |
| | B17"The hospital opened in 2022 and there was a change of management in 2023. We have a reduced team, in addition to lacking resources to fund this survey." – MA |
| | B18“Risk management was implemented just a year ago.” – CE |
| | B19“I would provide courses and training for the units’ NSP personnel.” – AM |
| | F1 "[We need to have] time" – MT |
| | F2 “I would like to have achieved it, but there are many things, and I can't do it” – PB |
| | F3 “We have the opportunity to dedicate ourselves much more to NSP” – PR |
| B. Relational Connections | None. |
| C. Communications | None. |
| D. Culture | B1 “We need to strengthen our culture and protocols.” – RN |
| | F1 “Encourage teams about the importance of promoting safe care, to have a culture of safety (it is important for: patients, family members, management teams and the institution).” – BA |

| | |
|---|---|
| 1. Human Equality-Centeredness | None. |
| 2. Recipient Centeredness | None. |
| 3. Deliverer Centeredness | None. |
| 4. Learning-Centered | B1 "Professionals don't adhere to the survey; they don't think it's important." – RN |
| E. Tension for Change | None. |
| F. Compatibility | None. |
| G. Relative Priority | B1 "Due to some internal demands, I was unable to administer the survey this year." – PB |
| | B2 "Difficulty in complying with the entire internal training schedule, delaying the survey. It has been extended to 2024." – MG |
| | B3 "We actually participate in 2022 and we will do this survey every 2 years according to one of the guidance emails sent". – RS |
| | B4 "[We can't adhere because we have another] service requirements related to the ONA accreditation process." – MT |
| | B5 "Yes, we had five external evaluation processes in the 2023 agenda, which prevented adherence to any other process." – PR |
| | B6 "The sector had to respond to infraction notices, organizing pending issues with a deadline for compliance by December." – RS |
| H. Incentive Systems | None. |
| I. Mission Alignment | None. |
| J. Available Resources | None. |
| 1. Financing | None. |
| 2. Space | None. |
| 3. Materials & Equipment | B1 "Another problem is that the internet does not work throughout the hospital, making it difficult to collect data on site" – RN |
| K. Access to Knowledge & Information | B1 "Provision of courses for institutions" – MT<br>B2 "I think it would be important to have more clarification about the survey and a better way to raise awareness among individuals." – PE |
| | F1 "Training for NSPs, because there are NSPs who need this training" – PB<br>F2 "Conduct training for teams" – PB |

| **IV. Individuals domain:** The roles and characteristics of individuals | |
|---|---|
| **Construct name** | **Participant's statements classified by the CFIR construct** |

| | |
|---|---|
| A. High– level leaders | B1 It would be important that senior hospital managers, and not the NSP, receive the “invitation” to participate in the evaluation.” – MG<br>B2 “New managers and many new employees who do not understand and have not undergone any training on the topics covered in the survey.” – PI<br>B3 “Meeting with hospital managers raising awareness about the importance of carrying out the assessment.” – PB<br>B4 “Managers’ commitment” – PE<br>B5 “Communicate the senior management of the hospitals so that they demand participation and get involved in the evaluation”. – RS |
| B. Mid-level leaders | B1 “Incipient support from health professionals and hospital sector coordinators.” – RN |
| C. Opinion leaders | None. |
| D. Implementation Facilitators | None. |
| E. Implementation Leads | The statements were classified into the Capacity and Motivation categories of the COM-B model. |
| Capability | B1 “Yes, I couldn’t understand how to enter the system to carry out the evaluation, consequently I couldn’t pass it on to the servers.” – RO |
| Motivation | B2 "It was really my fault for not paying attention to answer." – PB |
| F. Implementation Team Members | None. |
| G. Other Implementation Support | None. |
| H. Innovation Deliverers | F1 “The manager must formalize the request for completion in a mandatory manner by means of a letter or memorandum so that it can be “complied with”. – MT |
| I. Innovation Recipients | The statements were classified into the Capacity and Motivation categories of the COM-B model. |
| Capability | B1 “Difficulty in responding from professionals.” – PB<br>B2 “Employees having difficulty using electronic questionnaires.” – PI |
| Motivation | B3 “Resistance and disruptive behavior” – MT<br>B4 “Resistance from the team to participate in previous years, team overloaded by the lack of professionals” – MG<br>B5 “We are a large hospital and we constantly have to deal with overcrowding and lack of resources (including technological ones), which makes us have teams that are extremely resistant to change.” – PE<br>B6 “Low response rates in 2022.” – MG<br>B7 “Difficulty adhering to the survey in 2022.” – RN |

| V. **Implementation Process** domain: the activities and strategies used to implement the innovation | |
|---|---|
| **Construct name** | **Participant's statements classified by the CFIR construct** |
| A. Teaming | None. |
| B. Assessing needs | None. |
| 1. Innovation Deliverers | None. |
| 2. Innovation Recipients | None. |
| C. Assessing Context | None. |
| D. Planning | None. |
| E. Tailoring Strategies | None. |
| F. Engaging | The statements were classified into the Innovation Delivers subconstruct. |
| 1. Innovation Delivers | B1 "There was a lack of guidance/monitoring from our representatives in our state, to train, answer questions, help us in implementing this survey." – MA<br>B2 "I did not receive the email regarding the evaluation." – MG<br>B3 "I believe it should be better publicized." – MT<br>B4 "I did not receive any emails about this assessment, so I did not participate." – MA<br>B5 "We did not know." – RN<br>B6 "The person who took over the NSP didn't know." – PB |
| | F1 "Wide publicity". – PE<br>F2 "Wide dissemination by the competent services." – MT<br>F3 "I would like disclosure to begin at least six months before the deadline." – PR<br>F4 "Improve communication. My WhatsApp group is very participative, but it is not always possible to follow this communication channel. Communication via email is safer and more official." – PI<br>F5 "I would strengthen the partnership between state management and the NSP" – AP<br>F6 "I would publicize it in advance, create teaching materials to raise awareness." – PE<br>F7 "I would facilitate access and make the evaluation process more widely and clearly publicized." – MG |

| | |
|---|---|
| | F8 "Disclosure on social networks, by email" – PB<br><br>F9 "I would make greater publicity about the assessment of safety culture" – MA<br><br>F10 "I would promote it more widely, especially in small hospitals, which is my case." – MG<br><br>F11 "Advance notice of the period in which this evaluation will take place." –CE<br><br>F12 "I would make more disclosures about the assessment on social media portals related to patient safety." – RJ<br><br>F13 "Send physical notification" – RN<br><br>F14 "I would try in any way to alert hospitals about the importance of the participation of the NSP coordinator" – MA |
| 2. Innovation Recipients | None. |
| G. Doing | None. |
| H. Reflecting & Evaluating | F1 "Show assessment results more clearly after survey completion." – PE |
| I. Implementation | None. |
| J. Innovation | None. |
| K. Adapting | None. |
